# One Health responses to prevent the occurrence of rabies due to attacks by a rabid stray dog

**DOI:** 10.1002/vms3.986

**Published:** 2022-10-31

**Authors:** Yuehong Wei, Dapeng Li, Zhicong Yang, Kuncai Chen, Xinhong Pan, Jianmin Xu, Shouyi Chen

**Affiliations:** ^1^ Guangzhou Center for Disease Control and Prevention Guangzhou, Guangdong province China; ^2^ Huadu District Center for Disease Control and Prevention Guangzhou, Guangdong province China

**Keywords:** health policy, infectious diseases, One Health, public health, rabies

## Abstract

**Background:**

The number of human rabies cases caused by pet dogs in Guangzhou has been decreasing after years of comprehensive interventions. Consequently, attacks by stray dogs become a major issue in rabies control.

**Objectives:**

To share our experience of successfully dealing with rabies to provide some inspiration for prevention and control in countries and regions affected by it.

**Methods:**

A multidisciplinary One Health response was initiated to control this outbreak. Rabies virus was detected by PCR in the brain tissue of the associated stray dog. The sequences were aligned with reference sequences downloaded from GenBank using ClustalX. The maximum likelihood method implemented in MEGA 5.0 software package was used in a phylogenetic analysis of the aligned sequences.

**Results:**

Twelve patients with exposure to the stray dog were identified in the field investigation. Rabies vaccines and immunoglobulin were administered to all patients within 48 h. After 1 year of follow‐up, no exposed patients showed symptoms. Maximum likelihood analysis of the nucleotide sequences obtained from the PCR products indicated that the rabies virus in the dog was closely related to isolates from neighbouring provinces of Guangdong as well as those from surrounding countries of China.

**Conclusions:**

Multidisciplinary One Health intervention is effective not only in the control of rabies but also in rapid emergency responses to attacks by rabid stray dogs.

## Graphical abstract

In July 2017, 12 residents (3 males, 9 females) were bitten by the same rabid stray dog. With the One Health strategy action, no one occurred rabies after 1 year of follow‐up. To share our experience of successfully dealing with rabies to provide some inspiration for prevention and control in countries and regions affected by it.

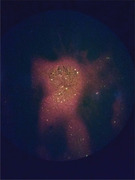

## 1 BACKGROUND

Rabies is caused by infection from a lyssavirus, which is a threat to humans and animals (Depani et al., 2012). All mammals are susceptible to rabies virus and may become a source of human infection. Human infection with rabies is caused by damaged skin or mucous membranes contaminated by saliva, secretions, and excretions of animals infected with rabies virus (Hemachudha et al., 2013). The clinical manifestations are mainly manic, and there is currently no effective treatment after the onset of symptoms. Once the clinical signs appear, the mortality rate is 100% (Fooks et al., 2014). Tens of thousands of cases of human rabies are reported every year worldwide, most of which occur in developing countries in Asia and Africa (Chen et al., 2017; Qi et al., 2018). In developing countries mammals that transmit rabies virus primarily include dogs, cats and wild animals, and domestic dogs are the main source of infection (Hueffer & Murphy, 2018; LeRoux et al., 2018). In developed countries where dog rabies has been under control, wild animals are the main sources of infection (Brunker & Mollentze, 2018; Chen et al., 2018; Gilbert et al., 2018).

Guangzhou, China, was once a city with a high prevalence of rabies. Before the 1990s, dozens of cases were reported each year (Wei et al., 2019). After years of effective management and continuous improvement of the monitoring system as parts of a multidisciplinary control strategy, the number of cases in Guangzhou has dramatically decreased since the 1990s. Like most countries or regions with a high incidence of rabies, domestic dog management is the focus of rabies epidemic control in Guangzhou. Although effective registration and immunisation have been established for pet dogs, the threat from stray dogs is not actively addressed. Rapid response to emergencies caused by stray dogs is a top priority before a management policy for stray dogs has been established.

In July 2017, a rabies outpatient human clinic in Huadu District, Guangzhou reported attacks by a suspected stray dog. We analysed the successful emergency responses by a multidisciplinary team. They can potentially serve as a reference for regions with similar cases to achieve the World Health Organization's goal of eliminating human rabies by 2030 (Minghui et al., 2018; Mohammadi, 2016).

## 2 METHODS

### 2.1 Research site

**FIGURE 1:**
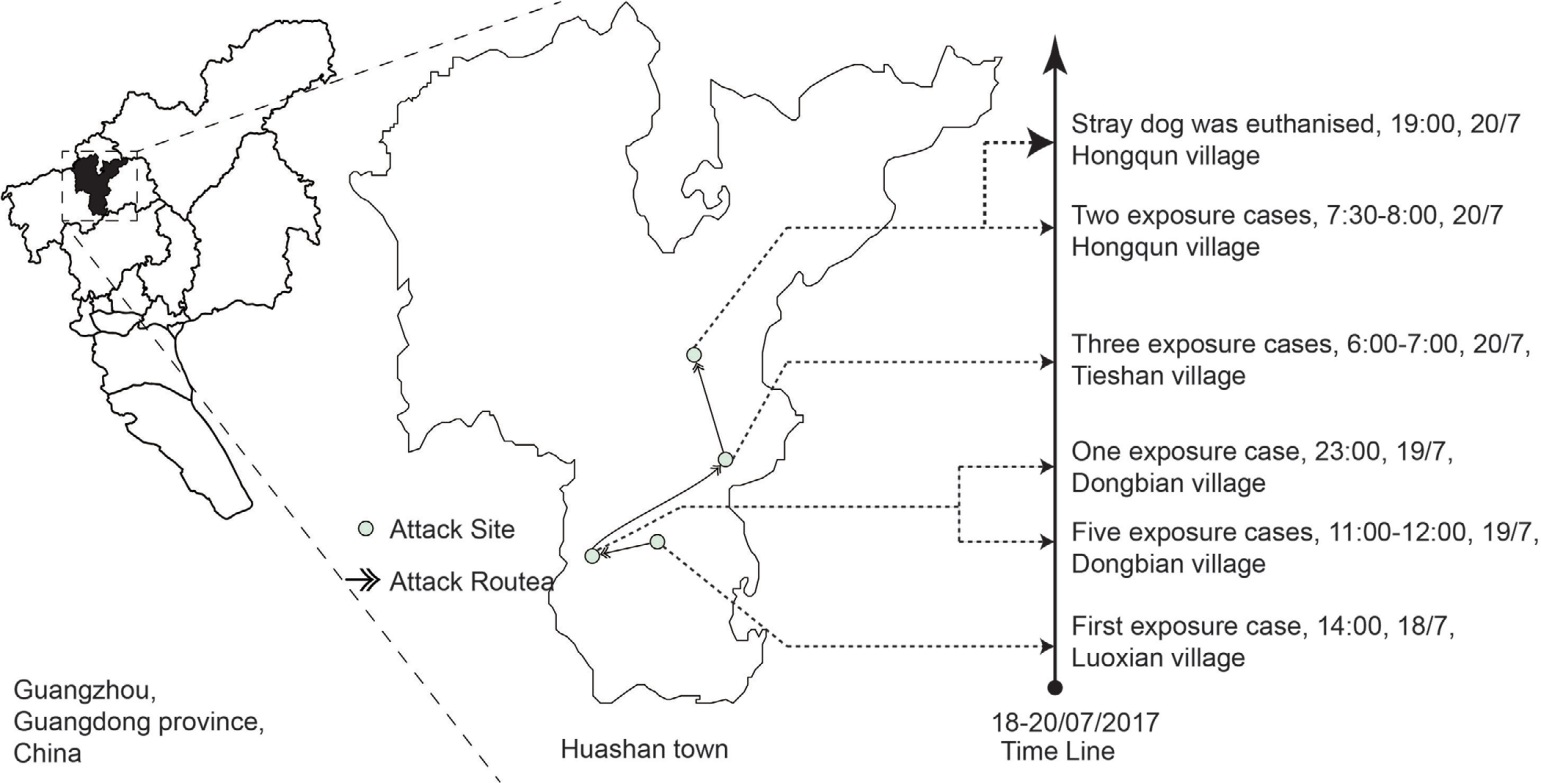
Location of Huashan town and the timeline of stray dog attacks

Huashan Town is in the Huadu District of Guangzhou City (Figure 1), with an area of approximately 116.4 km^2^ and a resident population of approximately 100,000. Huadu District was the hardest hit area of rabies epidemics before the 1990s, together with the adjacent Conghua District and Baiyun District. Although there have been no reports of rabies cases in Huadu District in recent years, the number of dog bites has increased each year, with stray dogs posing a huge threat to public safety.

### 2.2 Rabies monitoring management system

After years of exploration, Guangzhou has established a rabies surveillance management system at the Guangzhou Center for Disease Control and Prevention (CDC) with multidisciplinary collaboration from other agencies and partners (Wei et al., 2019). Hospitals at all levels and rabies outpatient clinics are responsible for monitoring, searching and follow‐up of exposed cases; public security is responsible for the issuance and management of dog licenses; the Agricultural Animal Husbandry Bureau is responsible for planning the immunisation of dogs; the Bureau of Industry and Commerce is responsible for the management of dog transactions; and local disease control centres and the government, resident committees and elementary and middle schools are mainly responsible for publicising the knowledge and planning activities to combat rabies infections.

The multidisciplinary communication and collaboration strategy was designed to address a wide range of diseases involving human, animal and environmental health, with a focus on zoonotic diseases. The strategy advocates public health communication and cooperation among departments such as veterinary medicine, environmental science, and the agricultural sector in responses to public health issues such as emerging infectious diseases and climate change.

### 2.3 Data sources and case management

As required by the rabies surveillance system, after the emergency, the local government immediately searched for cases exposed to stray dogs in rabies clinics throughout the district (scratched or bitten by stray dogs in Huashan Town during 18–20 July 2017), compared the characteristics of the dogs and time and location of the attack, and determined the number of exposed cases. Twelve exposed cases, two staff members who euthanised stray dogs, and two sample collectors were all vaccinated with the rabies vaccine and immunoglobulin at the rabies outpatient clinic. The time of vaccination was recorded by the clinic.

The diagnostic criteria for rabies were based on the WS 281–2008 standard issued by the Ministry of Health, China: a history of epidemiological exposure and manic (or paralytic) manifestations; the diagnosis is considered confirmed if samples are antigen‐positive or nucleic acid‐positive for the rabies virus or if rabies viruses can be isolated (National Health Commission of the People's Republic of China, 2008).

### 2.4 Detection method

The sera of the exposed cases were analysed using the virus fluorescent antibody virus neutralisation method (FAVN). The N and G genes of rabies virus were amplified by reverse‐transcription PCR of RNA extracted from the brain tissues of the stray dog (Feng et al., 2016), with products being sequenced.

The sequences were aligned with reference sequences downloaded from GenBank using ClustalX. The maximum likelihood method implemented in MEGA 5.0 software package was used in a phylogenetic analysis of the aligned sequences. The nucleotide sequences generated from the study were deposited in GenBank under accession numbers MK124757 and MK124758.

### 2.5 Event

In the morning of 20 July 2017, five people bitten by the same suspected rabid dog visited the rabies outpatient clinic of Huashan Hospital. After receiving the report, the Guangzhou CDC launched a joint prevention‐and‐control action, with the participation of the district CDC, Huashan Town Government, public security, animal husbandry bureau, and other departments.

In the Huashan district, each rabies outpatient clinic searched for exposed cases bitten by a suspected rabid dog with the same characteristics. The local public security department checked surveillance monitors and searched for the rabid dog and exposed cases. The animal husbandry, agriculture, and forestry departments searched stray dogs in the relevant areas. Pet dogs in this region (a 2.5 km radius of the tracks by the rabid dog) were immunised immunisation. Rabies immunoglobulin and vaccine were administered to all exposed cases, and their rabies neutralising antibody levels assessed. Propaganda on rabies prevention was distributed throughout the entire district, and the public was educated about how to respond to bitten or scratched by animals, including advice on visiting rabies outpatient clinics after potential exposure.

**FIGURE 2:**
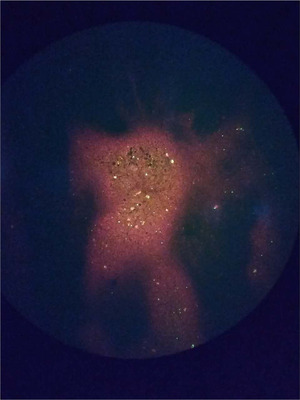
Positive for rabies virus in the stray dog brain tissue by fluorescent antibody test

The investigation identified the rabid dog as a stray dog. In the afternoon of 18 July, the first exposed case was attacked, followed by 6 people who were consecutively bitten by the same dog. In the morning of 19 July, another 5 people were attacked. On 20 July, the rabid dog was captured and euthanised (Figure 1), with brain tissue samples being collected. The results obtained in the afternoon of 21 July indicated that dog was positive (the FITC conjugate is Fujiribio, USA) for rabies virus (Figure 2).

## 3 RESULTS

### 3.1 General information on the exposed patients

A total of 12 exposed patients were identified (Table 1), including 3 males and 9 females, ranging in age from 7 to 82 years. Eleven patients were bitten on lower limbs, and 5 people had 3 wounds. The level of neutralising antibodies in all exposed patients reached the effective level (>0.5 IU/ml) after 5 doses of the vaccine (Liaoning Chengda Biological Products Co., Ltd.) and active immunotherapy (human rabies immune globulin, 20 IU/kg). During 1 year of follow‐up observations, no exposed patients showed symptoms.

**TABLE 1:** Information on the exposed cases

Number	Age/ gender	Attacked time	Bitten site/wounds’ number	Neutralising antibody before and after PEP*
1	82/F	02:00 PM/7‐18	Left lower limb/1	0.02/10.26
2	7/M	11:00 AM/7‐19	Both lower limbs/3	0.22/10.26
3	43/F	11:00 AM/7‐19	Right lower limb/1	0.22/7.79
4	8/F	11:00 AM/7‐19	Right lower limb/1	0.29/4.50
5	85/F	11:00 AM/7‐19	Left lower limb/ 1	0.29/>10.26
6	23/F	11:00 AM/7‐19	Right lower limb/1	0.13/5.92
7	47/M	11:00 AM/7‐19	Right lower limb/1	0.07/3.42
8	64/F	06:40 AM/7‐20	Both lower limbs/3	0.22/>10.26
9	33/F	06:30 AM/7‐20	Right lower limb/3	0.02/4.5
10	55/F	06:10 AM/7‐20	Left lower limb/2	0.02/7.79
11	54/F	06:45 AM/7‐20	Left lower limb/3	>10.26^§^/>10.26
12	52/M	06:50 AM/7‐20	Left lower limb/3	>10.26^§^/>10.26

* The effective level of neutralising antibody is > 0.5 IU/ml.

^§^ High level of neutralising antibody before PEP probably because vaccinated years ago.

### 3.2 Homology analysis of rabies viral G and N gene

**FIGURE 3:**
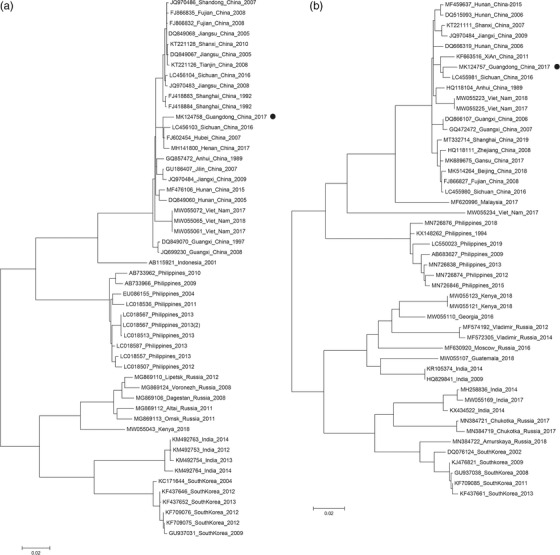
3A‐ML Phylogenetic tree‐G gene 3B‐ML Phylogenetic tree‐N gene

The G and N gene sequences of the rabies virus obtained from the rabid dog in Guangdong were compared with those from rabies virus in adjacent provinces and neighbouring countries of China. The phylogenetic tree constructed with the maximum likelihood method showed that the Guangzhou rabies virus strain was located on the same branch as the strains of the neighbouring provinces. In addition, it was closely related to the strains from neighbouring countries (Figure 3a and b).

## 4 DISCUSSION

### 4.1 Critical elements for successful handling of rabies emergencies

The monitoring network of rabies outpatient clinics as the sentinel has played an important role in the identification of this public health emergency. When abnormally exposed patients (bitten or scratched by animals) present to the hospitals, they are required by the network policy to receive close attention, with the intention to identify the causes. Furthermore, the cases should be properly handled and treated to prevent the occurrence of serious public health incidents. The rabies surveillance and management system involves multiple public health and safety sectors and agencies and is based on the One Health strategy. The previous human‐oriented prevention and control strategy has been transformed into a human‐ and animal‐specific prevention and control system involving promotion of dog vaccinations, control of stray dogs, removal of infection sources and post‐exposure vaccination and antiserum treatment of patients, and public education. After years of practice and improvement and formal implementation, there have been no reported cases of human rabies in Guangzhou during the past 2 years, which has demonstrated the effectiveness of this control measure. Among them, the extensive educational activities on rabies prevention and control is a prerequisite for the successful outcome in this event; through organised events in elementary and middle schools, community committees, advertisements in television and network media, educational leaflets, and World Rabies Day, Guangzhou has educated the public regarding the seriousness of rabies, major transmission routes, the importance of preventive vaccination and immunoglobulin administration immediately after exposure, and the role of regular vaccination of pet dogs.

### 4.2 Human disposal of stray dogs

There are tens of thousands of stray dogs in Guangzhou today, most of which are abandoned. Although there are more than a dozen civilian animal shelters and rescue stations, because of the large number of stray dogs, this is still a drop in the bucket. In 2013, the Guangzhou Municipal Public Security Bureau established the Guangzhou Dog Shelter and Inspection Office to receive, inspect, and process abandoned, stray, detained, and confiscated dogs, with a focus on advocating civilised, scientific dog‐keeping and animal protection. In terms of reducing the number of stray dogs, the effects have been minimal. In many countries with rabies epidemics, stray dogs are a major public health hazard. Several countries, including India, have implemented the Animal Birth Control (ABC) program, which has achieved good outcomes by vaccinating and sterilising stray dogs to control their numbers and rabies (Totton et al., 2011). Research from Sri Lanka shows that the ABC project is far from sufficient to eliminate threats. According to the density, population structure, and living range of stray dogs and pet dogs in different human environments, government departments should work with veterinarians to develop better strategies (Hou et al., 2012). Countries such as Japan, Thailand and the Philippines have introduced regulations to eliminate stray dogs and register, manage and strengthen the immunisation of pet dogs, Guangzhou has established several regulations on pet dogs, but control strategy for stray dogs is still being studied.

### 4.3 The importance of PEP (post‐exposure prophylaxis)

Rabies is a zoonotic disease that can be prevented but not cured. Vaccination and immunoglobulin administration are the only effective post‐exposure measures to prevent rabies. Most national strategies for the prevention and control of human and animal rabies are based on active vaccination, including large‐scale immunisation against domestic animals, mainly dogs, and wild animals such as foxes and raccoons, in addition to the post‐exposure immunisation of humans. Therefore, the supply of high‐quality human and animal rabies vaccine, economical and convenient PEP treatment after exposure, and high public awareness of rabies prevention are the first critical steps to eliminate rabies.

### 4.4 Rabies control in the era of globalisation

Results of the phylogenetic analysis indicate that rabies virus strains in Guangzhou are closely related to the strains in neighbouring provinces and countries, highlighting the difficulty of rabies elimination in the era of globalisation (Chen et al., 2017; Lankau et al., 2012). In areas where canine rabies virus has been eliminated, globalisation may lead to rabies viruses being imported and the re‐emergence in domestic animals, with serious consequences. To prevent major rabies events caused by globalisation, international collaborations by public health, animal health, tourism, and custom inspection and quarantine departments are needed. They should work together using the One Health strategy in preventing and controlling rabies virus exports and imports (Fitzpatrick et al., 2016; Perez de Diego et al., 2015; Rock et al., 2017; Tan et al., 2017).

## 5 CONCLUSION

In an area where human rabies has not been eliminated, the rapid and effective management of attacks by rabid stray dog requires coordinated collaborations of public health, safety and agricultural agencies and local governments. The One Health strategy is effective in both rabies control and prevention and management of post‐exposure rabies emergencies. Today, with increased globalisation, we should strive to share successful experiences and seek international cooperation to eliminate human rabies.

## AUTHOR CONTRIBUTIONS

Yuehong Wei: conceptualisation, writing‐original draft, writing‐review & editing. Dapeng Li: conceptualisation, writing‐original draft, writing‐review & editing. Zhicong Yang: data curation, funding acquisition, project administration, supervision. Kuncai Chen: data curation, project administration, supervision. Xinhong Pan: data curation, methodology. Jianmin Xu: data curation, investigation. Shouyi Chen: project administration, resources, supervision, validation, writing‐review & editing.

### ETHICAL STATEMENT

This study was approved by the Ethics Committee of Guangzhou Center for Disease Control and Prevention (GZCDC).

### PEER REVIEW

The peer review history for this article is available at https://publons.com/publon/10.1002/vms3.986.

## Data Availability

The data that support the findings of this study are available from the corresponding author upon reasonable request.
